# Editorials

**Published:** 1911-07

**Authors:** 


### EDITORIALS

The Business Office of the *Journal-Record* is 1 1-2 to 5 1-2 South Broad Street.
The Editorial Office is 1014-15 Century Building.
Address all Business Communications to *Journal-Record of Medicine*, 1 1-2 to 5 1-2 South Broad Street.
Make remittances payable *to The Journal-Record of Medicine.*

On matters pertaining to the Editorial and Original Communications, address Edgar G. Ballenger, M. D., Atlanta, Ga.
Reprints of Original Articles will be furnished at cost price. Requests for the same should always be made in THE MANUSCRIPT.

We will present, postpaid, on request, to each contributor of an original article, twenty (20) marked copies of *The Journal-Record of Medicine* containing such article.

### TYPHOID FEVER.

Beginning in this issue the Journal-Record will present a series of papers upon typhoid fever. These papers comprise the symposium upon this subject which was read at the two special meetings recently held in accord with the tentative "post-graduate plan" suggested as a partial substitute for the customary program of the Fulton County Medical Society. The interest manifested and the success of these two meetings would seem to indicate that perhaps in the future it might not be a bad plan to have every other meeting of this character. The program committee, headed by Dr. Willis Westmoreland, is due much credit for the careful subdivision and assignment of the subject. We are under many obligations also to those who took so much care in preparing the papers upon the various sub-heads.

There are many attractive features about these special meetings and not the least of them is the informal Dutch supper which follows and affords an excellent opportunity for the physicians to meet and make new or renew old acquaintances. We heartily endorse the new plan and believe it is a step in the right direction.

### THE CHATTAHOOCHEE VALLEY MEDICAL ASSOCIATION.

Unusually interesting and pleasant was the last meeting of the Chattahoochee Valley Medical Association, held July 18th and 19th at Tuskegee. The somewhat troublesome railway schedules made the attendance less than it should have been, but the quality of the work and the enthusiasm shown more than compensated for the only average crowd. Rarely, if ever before, has the association been more cordially entertained or received more courteous attention both from the local physicians as well as the lay citizens, to whom the association is under many and lasting obligations. We received a rare treat of old-fashioned "Southern hospitality," for which our Southland was once so noted, but now, owing to its rapid commercial development, it has almost relegated to the past. A notice regarding this meeting would be incomplete were we to omit reference to the pleasant impressions received from the visit to the Tuskegee Institute, for which Booker Washington has worked so faithfully and so successfully. One cannot see it without feeling a higher regard for its founder.

The Chattahoochee Valley Medical Association is a wide-awake society, interested chiefly in the scientific and social side of medicine and fortunately is not burdened with an incubus of politics and business. The next meeting will be held in La Grange, a point easily reached by a large number of physicians who are and who should be members. The Journal-Record would earnestly request a large attendance, as the meetings are really worth while and few can afford to miss them.

